# A DNA Sequence Recognition Loop on APOBEC3A Controls Substrate Specificity

**DOI:** 10.1371/journal.pone.0097062

**Published:** 2014-05-14

**Authors:** Eric C. Logue, Nicolin Bloch, Erica Dhuey, Ruonan Zhang, Ping Cao, Cecile Herate, Lise Chauveau, Stevan R. Hubbard, Nathaniel R. Landau

**Affiliations:** 1 Department of Microbiology, NYU School of Medicine, New York, New York, United States of America; 2 Kimmel Center for Biology and Medicine of the Skirball Institute, NYU School of Medicine, New York, New York, United States of America; 3 Department of Biochemistry and Molecular Pharmacology, NYU School of Medicine, New York, New York, United States of America; French-German Advanced Translational Drug Discovery Center, France

## Abstract

APOBEC3A (A3A), one of the seven-member APOBEC3 family of cytidine deaminases, lacks strong antiviral activity against lentiviruses but is a potent inhibitor of adeno-associated virus and endogenous retroelements. In this report, we characterize the biochemical properties of mammalian cell-produced and catalytically active *E. coli*-produced A3A. The enzyme binds to single-stranded DNA with a K_d_ of 150 nM and forms dimeric and monomeric fractions. A3A, unlike APOBEC3G (A3G), deaminates DNA substrates nonprocessively. Using a panel of oligonucleotides that contained all possible trinucleotide contexts, we identified the preferred target sequence as TC (A/G). Based on a three-dimensional model of A3A, we identified a putative binding groove that contains residues with the potential to bind substrate DNA and to influence target sequence specificity. Taking advantage of the sequence similarity to the catalytic domain of A3G, we generated A3A/A3G chimeric proteins and analyzed their target site preference. We identified a recognition loop that altered A3A sequence specificity, broadening its target sequence preference. Mutation of amino acids in the predicted DNA binding groove prevented substrate binding, confirming the role of this groove in substrate binding. These findings shed light on how APOBEC3 proteins bind their substrate and determine which sites to deaminate.

## Introduction

APOBEC3A (A3A) is one of the seven-member APOBEC3 family of cytidine deaminases encoded in the human genome [1]–[3]. The protein has potent cytidine deaminase activity but unlike other family members, such as APOBEC3F (A3F) and APOBEC3G (A3G), does not appear to have considerable activity against HIV-1. A3F and A3G are packaged in the viral core as the virus assembles, most likely through interactions with the viral genomic RNA in a complex with the nucleocapsid protein of Gag [4]–[14]. Upon reverse transcription, the packaged enzymes deaminate the minus-strand of the viral reverse transcript, resulting in G→A mutation upon synthesis of the plus strand [15]–[19]. The lentivirus accessory protein Vif counteracts A3F and A3G by binding to and promoting their degradation [20] but fails to bind and induce A3A degradation. A3A can be packaged into HIV-1 virions but lacks antiviral activity toward HIV-1 and SIV [21]. Given A3A cytidine deaminase activity and its ability to be packaged, it is unclear why it lacks antiviral activity. Some reports suggest that it inefficiently packages or that it is packaged such that it cannot access the reverse transcription complex. Fusion of A3A to Vpr or the N-terminus of A3G activates the antiviral activity of the protein, perhaps by causing it to associate more tightly with the virion core [22], [23].

Expression of A3A is largely restricted to myeloid cells such as monocyte-derived macrophages (MDM) and monocyte-derived dendritic cells (MDDC). The expression of A3A is upregulated in these cells in response to type I interferon treatment, which suggests a role for A3A in the antiviral response [24]–[26]. While A3A has little activity against lentiviruses, it potently inhibits the parvoviruses, adeno-associated virus (AAV) and minute virus of mice (MVM) [23]. It also has strong activity against human T-lymphotropic virus type I (HTLV-1) [27] and endogenous retroelements [21], [28], [29]. The mechanism by which A3A restricts AAV and retroelements is unclear. The inhibitory activity of A3A against both elements is dependent on amino acids in the catalytic active site yet no increase in the frequency of C→T or G→A mutations were detected. Moreover, a mutated A3A that lacks catalytic activity retains activity against AAV [28]. In addition to its antiviral role, A3A was reported to clear foreign DNA from the cytoplasm of monocytes in a cytidine deamination-dependent manner [26]. This function might explain how A3A restricts HIV-1 in macrophages even though it does not appear to mediate cytidine deamination of the HIV-1 genome when packaged into the virus [24], [25], [30], [31].

A3A, like all APOBEC3 family members, specifically deaminates single-stranded DNA (ssDNA). It consists of a single deaminase domain, a feature in common with APOBEC3C and APOBEC3H but different from APOBEC3B, APOBEC3D, A3F, and A3G which have two deaminase domains. When expressed in myeloid cells, A3A localizes in the cytoplasm and is not genotoxic [32]. However, upon ectopic expression, A3A localizes to both the cytoplasm and the nucleus and causes DNA damage [33]. This localization to both the cytoplasm and nucleus is in contrast to A3F and A3G, which are largely cytoplasmic. A3G acts as a dimer to processively deaminate ssDNA [34], while a recent study suggests that A3A does not deaminate processively and functions as a monomer [35]. A3A has sequence homology to the carboxy-terminal domain of A3G [2] and mutagenesis studies identified several conserved amino acids that are critical for deaminase activity in both A3A and A3G [36]. The initial characterization of A3A identified (T/C) CA as the preferred trinucleotide motif for deamination [21], which contrasts with the CCC trinucleotide preferred by A3G. How the sequence differences between A3A and A3G account for this altered sequence specificity has not yet been determined.

Here, we biochemically characterize the enzymatic activity of catalytically active A3A produced in *E. coli* and in mammalian cells. We found that the protein is a dimer that acts nonprocessively with a preference for a TC (A/G) trinucleotide. By generating chimeric proteins in which loops of A3A were exchanged with those of A3G, we identified a putative sequence recognition loop, which determines the deamination target site specificity. We also identified several residues in the putative ssDNA binding groove and a second putative recognition loop that were required for A3A interaction with ssDNA and identified two amino acids that are specific to A3A that appear to play a role in reducing the affinity of the protein for ssDNA.

## Results

### A3A Forms Multimers

**Figure 1:**
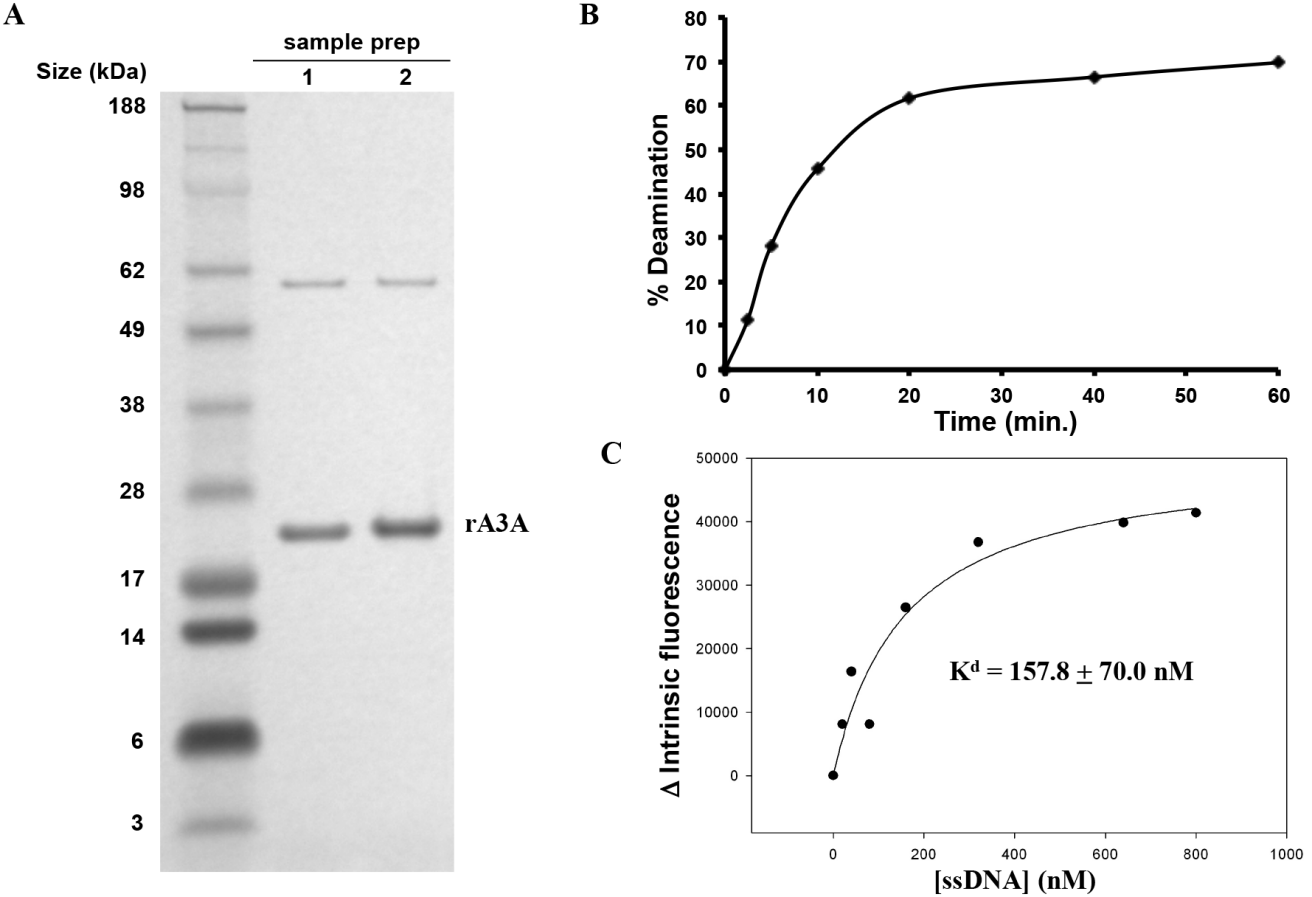
rA3A produced in *E. coli* binds and deaminates ssDNA. A) rA3A was visualized by Coomassie staining B) The extent of deamination was determined for rA3A incubated with ssDNA for 2, 5, 10, 20, 40, or 60 minutes. C) DNA binding was determined by measuring the change in the intrinsic fluorescence of rA3A following incubation with an increasing amount of ssDNA.

**Figure 2:**
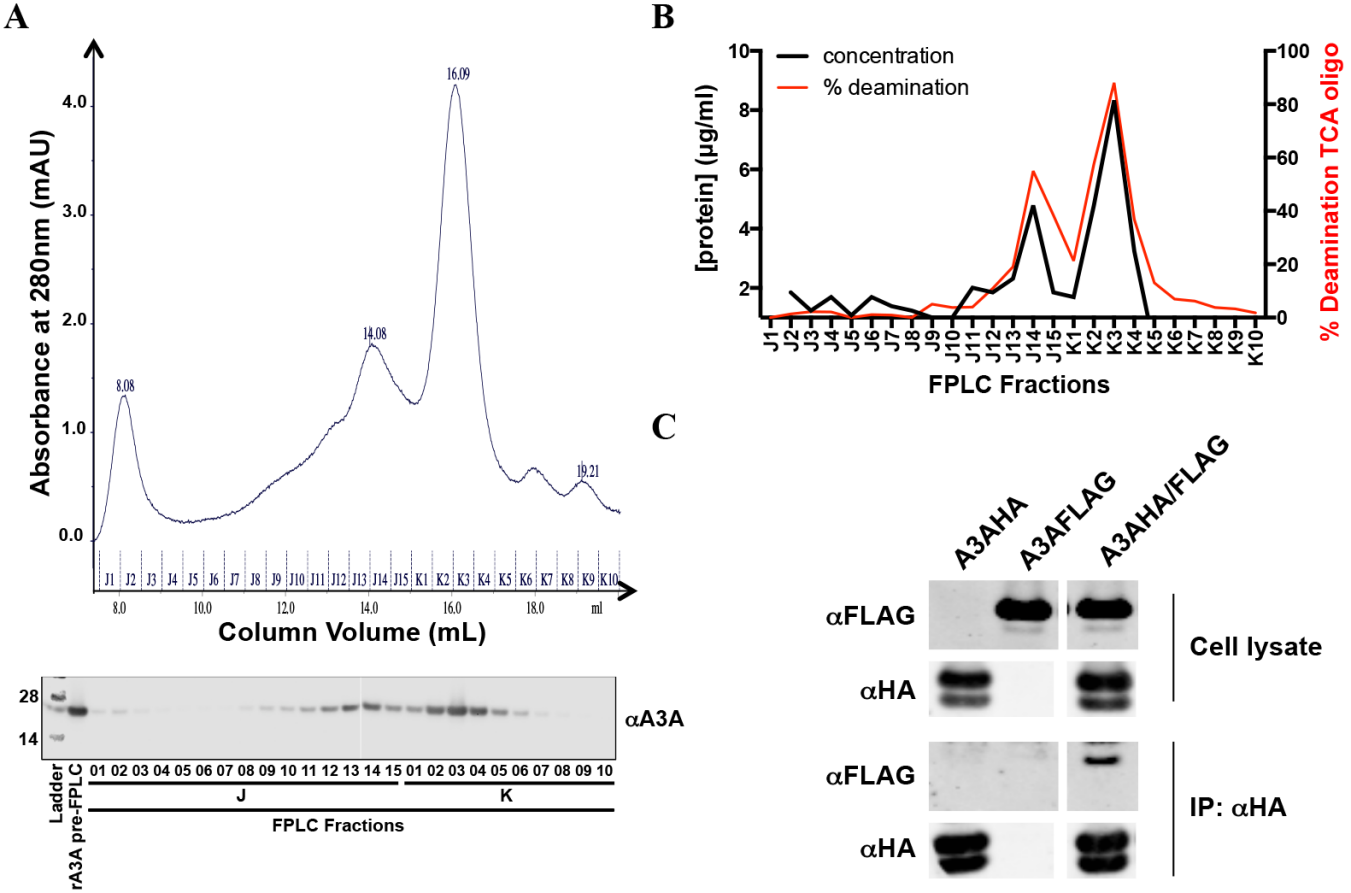
A3A homodimerizes *in vitro* and *in vivo*. A) *top.* rA3A was run on a size exclusion column. *bottom.* A3A content was tested for each fraction by immunoblot using the anti-A3A antibody. B) The overall protein content of each fraction was determined by Bradford assay, and catalytic activity was determined by *in vitro* deaminase assay with a biotinylated TCA oligo using an equal volume of each fraction. C) 293T cells were transfected with expression vectors for HA, FLAG-tagged A3A or both. The cells were lysed, and the HA-tagged A3A was immunoprecipitated. The immunoprecipitates were then analyzed on an immunoblot.

To biochemically characterize A3A, we generated highly purified recombinant A3A (rA3A) in *E. coli* (Fig. 1A). The rA3A was expressed as a GST fusion protein, affinity purified using glutathione beads and then released from the matrix by cleavage with factor Xa. In the preparation of the protein, we found that its expression was highly toxic to the bacteria and that upon induction it induced G→A mutations in the expression vector used to express it. We found, however, that minimizing the induction phase allowed us to produce large quantities of pure protein. The resulting protein was catalytically active since it retained its ability to deaminate oligonucleotide targets in a deaminase assay (Fig. 1B). It also retained its ability to bind to ssDNA targets with a calculated K_d_ of 158±70 nM (Fig. 1C). To determine the dimerization state of A3A, we subjected the rA3A to size exclusion chromatography (SEC). Analysis of the fractions showed that rA3A ran as both a monomer and a dimer in the buffer used for measuring deaminase activity (Fig. 2A). While the majority of rA3A appears to be monomeric, there is a significant fraction that exists as a dimer. Both monomeric and dimeric fractions displayed high deaminase activity (Fig. 2B and Fig. S1).

Since the production of A3A in bacteria or conditions used during the SEC might have led to the observation of artifactual multimers [37], we asked whether such multimers could be observed upon expression of A3A in mammalian cells. We therefore cotransfected 293T cells with expression vectors for HA and FLAG-tagged A3A. We then immunoprecipitated with anti-HA antibody and analyzed the proteins on an immunoblot probed with anti-FLAG antibody. This analysis showed that FLAG-tagged protein coimmunoprecipitated when expressed with A3A-HA, demonstrating that A3A forms oligomers in the cell (Fig. 2C), although it does not distinguish dimers from higher order multimers.

### A3A Deamination is Nonprocessive

**Figure 3:**
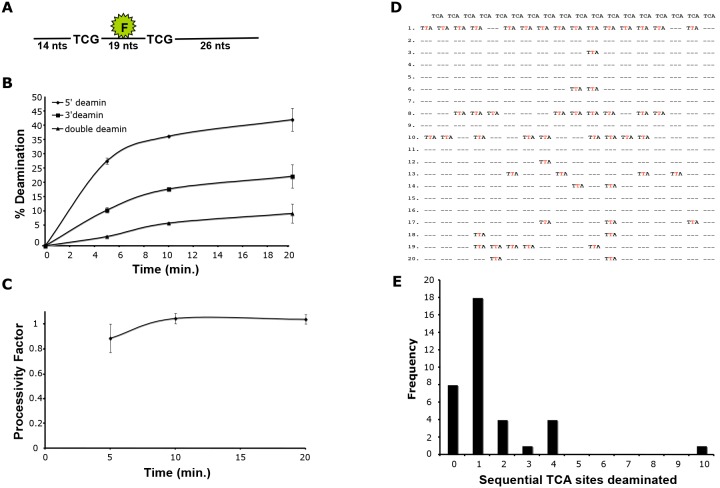
A3A deamination is nonprocessive. A) An oligonucleotide containing a fluorescein tag flanked by consensus deamination sites was incubated with rA3A. B) The oligonucleotide was incubated with rA3A for 2, 5, 10 and 20 minutes, and single or double deamination at the 5′- and 3′-target sites was determined. C) The processivity factor was calculated for each time point. D) An oligonucleotide containing a series of target sites was incubated with rA3A and deamination states were identified by DNA sequencing. E) The frequency of deaminations at sequential target sites were quantified. Eight of the sequenced oligonuleotides contained no mutations and are indicated as having 0 sequential deamination sites.

A3G processively deaminates ssDNA. To determine whether A3A also deaminates processively, we established a deaminase assay using an oligonucleotide substrate containing a fluorescein tag flanked by two TCG trinucleotides (Fig. 3A). This assay allowed us to determine whether deamination occurred at the 5′ deamination site, the 3′ deamination site or at both sites. We found that rA3A preferentially deaminated the 5′ site (Fig. 3B). Using these data, we calculated the processivity factor, a measure of whether double deamination occurs more frequently than that predicted by the deamination frequency at the two independent deamination sites. The calculated processivity factor was less than or equal to one at all time points tested, indicating that double deamination occurred at the frequency expected for the two independent sites. This suggests that A3A deaminates ssDNA using a nonprocessive mechanism (Fig. 3C).

We further tested processivity by determining the ability of rA3A to deaminate an oligonucleotide substrate containing deamination sites arrayed in tandem. Following this deamination reaction, the oligonucleotide substrate was subjected to one round of amplification, cloned into TOPO-TA vector and the resulting clones were sequenced (Fig. 3D). Eight of the twenty sequenced clones had no deaminations (0 sequential deamination sites). The clones that had deamination sites showed little evidence of sequential deamination since most of the deaminated sites lacked adjacent deaminated sites (Fig. 3E). The few sites that did have sequential deamination sites generally had no more than four sites sequentially deaminated. These data further suggest that A3A utilizes a nonprocessive deamination mechanism.

### A Putative Recognition Loop Constrains A3A Sequence Specificity

**Figure 4:**
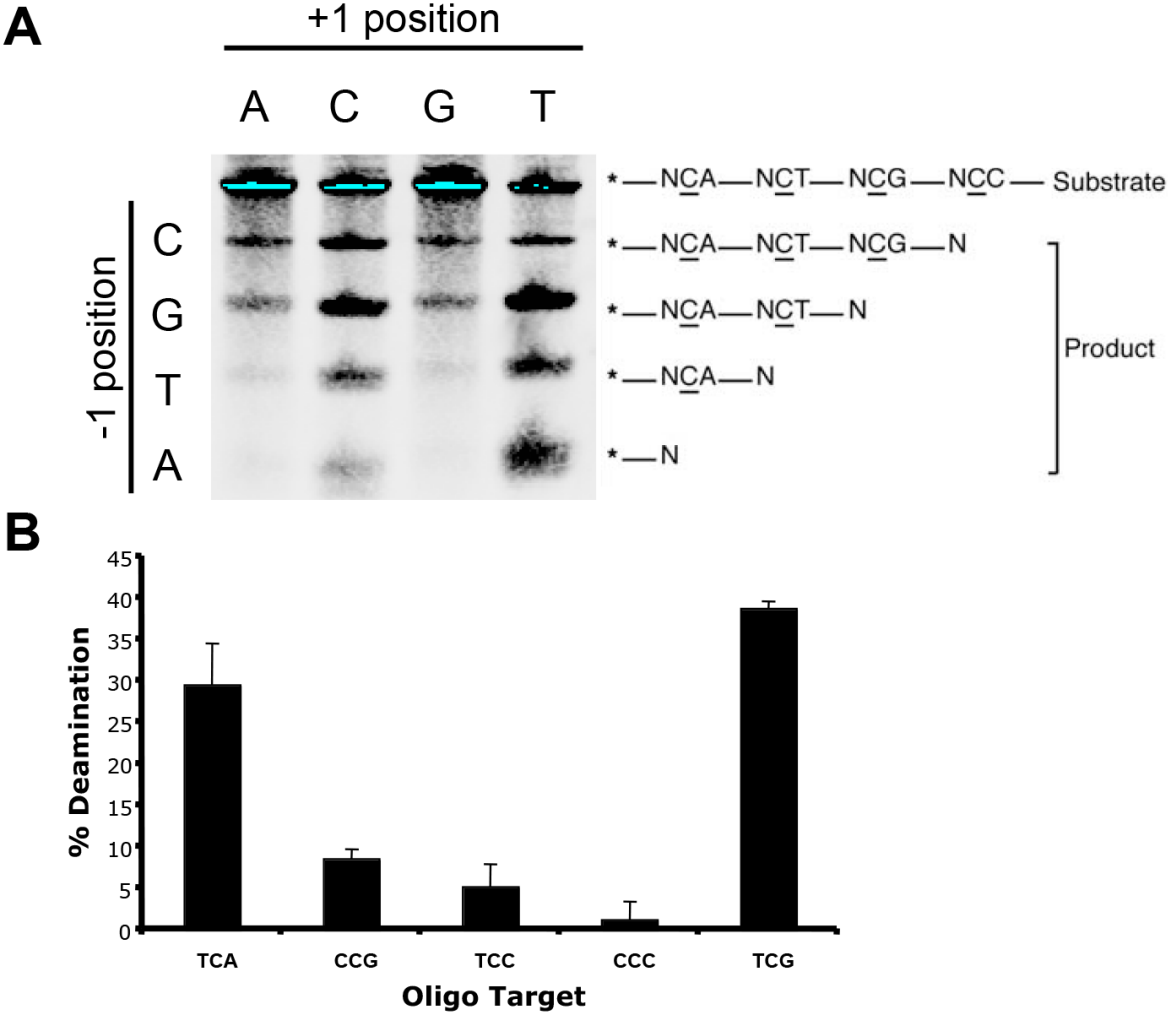
The preferred consensus deamination target site of A3A is TC (A/G). A) rA3A was incubated with ssDNA substrates containing four different target site sequences. B) rA3A was incubated with ssDNA substrates containing different target site sequences.

We used a panel of four oligonucleotides to determine the target site preference of A3A, each of which contained four tandem deamination sites. The four 5′-biotinylated oligonucleotides varied in the nucleotide 5′ of the target cytosine (N, different for each oligo), and each oligo contained four different deamination sites differing at the nucleotide 3′ of the target cytosine (NCA, NCT, NCC, and NCG). Each oligonucleotide was incubated with rA3A, and the products were separated on a gel and analyzed as in the *in vitro* deaminase assay. The analysis demonstrated that rA3A preferred a TC dinucleotide (Fig. 4A). Further deamination experiments using oligonucleotide substrates containing a single deamination site showed that TC followed by A or G was the preferred trinucleotide motif (Fig. 4B). Thus, the consensus target for A3A is TC (A/G). These results are in agreement with a previous study investigating the 5′ context constraints of the deamination site [35] yet also describe specificity of A3A toward the 3′-nucleotide context as well.

**Figure 5:**
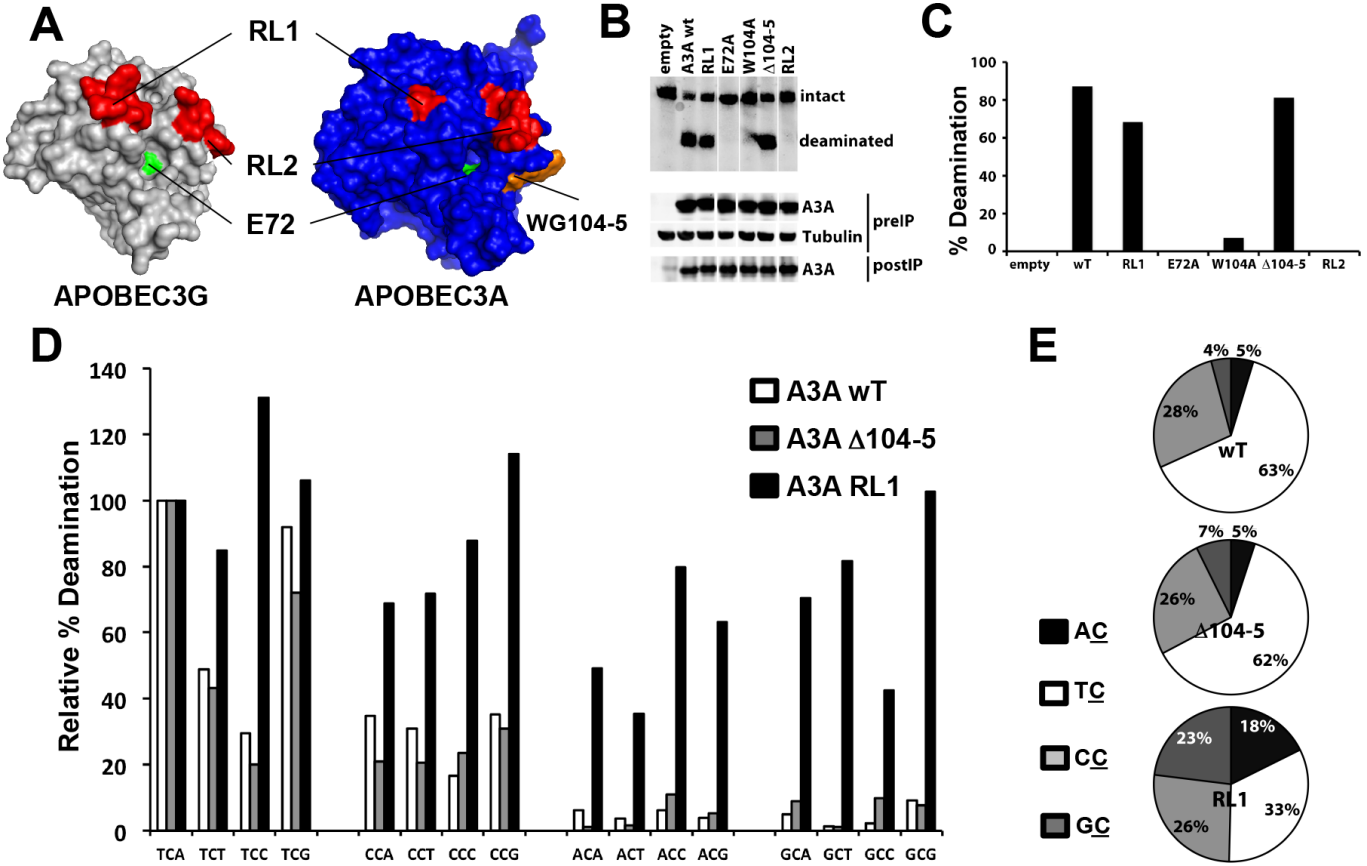
The target site preference of A3A is influenced by RL1. A) A model of A3A on the A3G crystal structure shows loops RL1 and RL2 (in red) flanking the proposed DNA binding groove and shows the inserted the WG amino acids (in orange) close to the conserved catalytic E72 (in green). Mammalian expression vectors for A3A in which RL1 or RL2 of A3G was swapped into A3A (RL1 and RL2) and a mutated A3A deleted for the WG were generated. B) The mutated proteins were expressed in transfected 293T cells, immunoprecipitated from cell lysates and then tested for deaminase activity against an oligonucleotide containing a TCG target sequence. C) The deamination activity of the mutated proteins was expressed as the ratio of the intensity of the deaminated product to the sum of the intensities of the unmodified and deaminated species. D) The target site specificity of the immunoprecipitated proteins was determined by incubating with oligonucleotides containing an NCN target site. The relative percentage deamination was determined as the percent deamination of the NCN oligonucleotide normalized to the TCA oligonucleotide. E) The target site specificity was determined as the ratio of deaminase activity determined for the NCs oligonucleotides to the total deamination activity.

The TC (A/G) consensus deamination site of A3A differs from the CCC consensus site of A3G. The catalytic domains of A3A and A3G are very similar in amino acid sequence and thus, relatively few amino acids are likely to determine this sequence preference. In order to test this hypothesis, we compared the sequences and structures of A3A and A3G (Fig. 5A and Fig. S2). To understand which amino acids might play a role in determining the sequence preference of the enzymes, we compared the NMR structure of A3A [38] to the crystal structure of A3G [39]. The comparison showed differences in the two loop regions located adjacent to the likely substrate-binding groove. These could be substrate recognition loops. In addition, A3A has a tryptophan and glycine residue (WG104-5) that is absent from A3G. The A3A NMR structure shows that the side-chain of W104 points into the substrate pocket and thus might influence substrate specificity (Fig. 5A and Fig. S2).

To determine whether these differences account for the substrate specificities of A3A and A3G, we generated chimeric proteins in which recognition loop 1 (RL1) and recognition loop 2 (RL2) in A3A were replaced by the corresponding recognition loops of A3G. In addition, we generated A3A with a W104A mutation or a deletion of W104 and G105 (ΔWG104-5). To determine the catalytic activity of the mutated enzymes, we expressed them in transfected 293T cells, immunoprecipitated them, and tested the catalytic activity of the proteins in an *in vitro* deaminase assay. The results showed that the RL2 A3A and W104A mutants of A3A were inactive. In contrast, RL1 and ΔWG104-5 A3A retained activity against a TCA oligonucleotide (Fig. 5B and 5C). There was the possibility that the mutated proteins changed their target sequence preference. We tested the RL1 and ΔWG104-5 A3A for the ability to deaminate oligonucleotides containing every combination of trinucleotides. These results showed that the mammalian cell-expressed wild-type A3A had the same site preference as the E. coli-produced enzyme. ΔWG104-5 A3A maintained the preference for TC (A/G). In contrast, the RL1 chimera was active, but was more flexible with respect to the nucleotide 5′ to the deaminated C, deaminating AC, CC and GC with increased frequency (Fig. 5D and 5E). These data suggest that the presence of GI25-26 in RL1 of A3A specifies the 5′ nucleotide while swapping the EPWVR residues from RL1 of A3G relieves this constraint.

### Mutational Analysis Confirms the Role of the Putative DNA Binding Site on A3A

**Figure 6:**
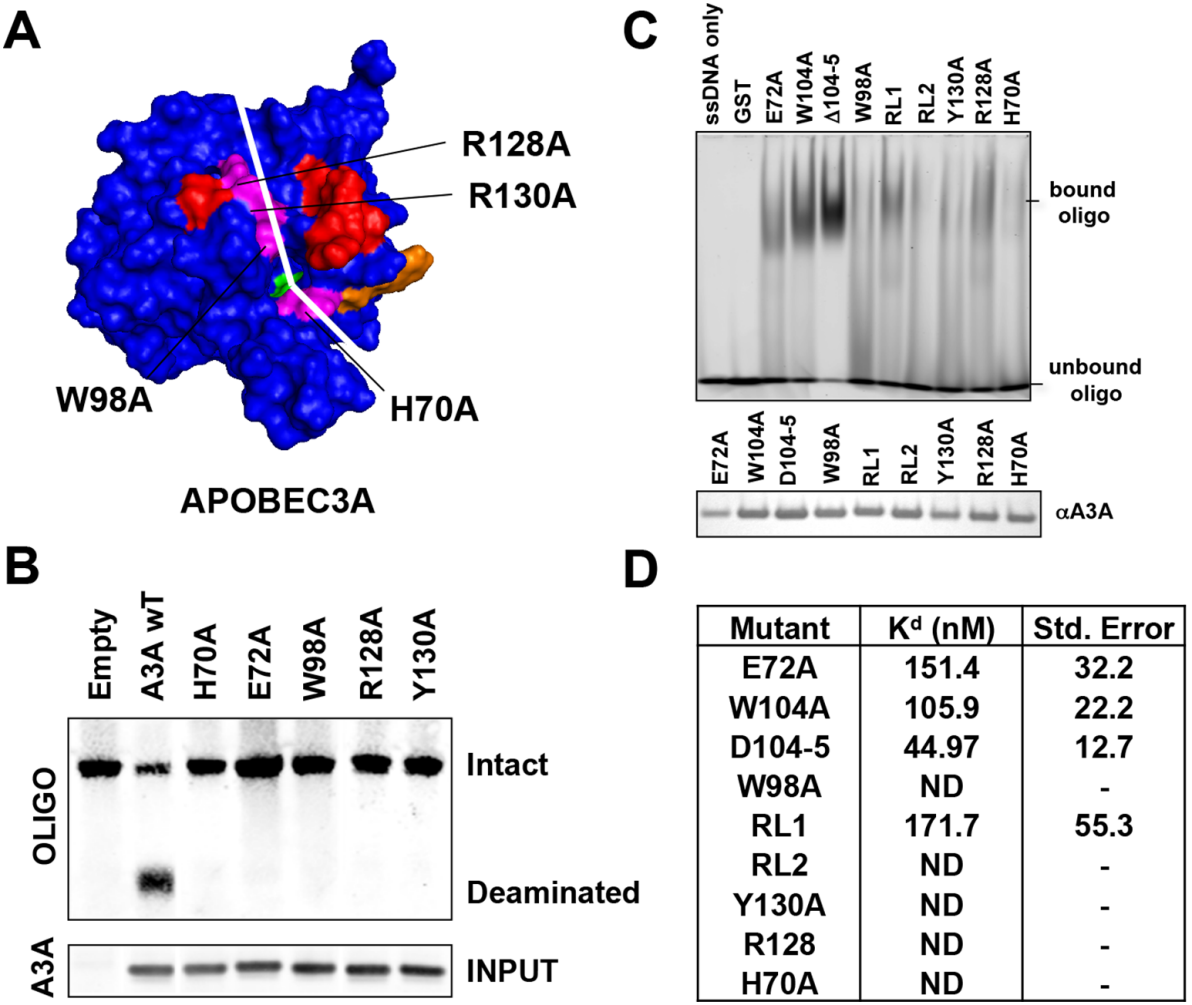
Residues in the putative DNA binding cleft determine binding to ssDNA. A) The A3A model predicts four conserved amino acids that could play a role in DNA binding. Each was mutated to alanine. B) The mutated A3A proteins were expressed in transfected 293T cells, immunoprecipitated from cell lysates and tested for deaminase activity against a deoxyoligonucleotide DNA containing a TCG target site. C) *E. coli* expressed mutated A3A proteins in the background of the E72A catalytic site mutation were purified and similar amounts of each were analyzed for ssDNA binding in the gel retardation assay. D) A fluorescein tagged oligonucleotide was incubated with increasing amounts rA3A from (C). The change in the extrinsic fluorescence at each protein concentration was used to fit a one-site saturation ligand-binding curve. The calculated K_d_ for each curve is displayed on a table together with the standard error. ND indicates that a K_d_ could not be determined.

The crystal structure of A3G shows a shallow groove in A3G that surrounds the catalytic core and is postulated to be the binding site for ssDNA [40]. A potential DNA-binding groove is also apparent in the NMR structure. We identified residues H70, W98, R128 and Y130 at the base of the groove as having the potential to interact with a DNA substrate (Fig. 6A). To test whether these amino acids are required for DNA binding, we generated alanine point mutations and tested their catalytic activity in the *in vitro* deaminase assay (Fig. 6B). The results showed that each of these residues was required for deaminase activity.

Because these residues surround the catalytic core, it is possible that the loss of deaminase activity was due to alterations in catalytic function rather than an inability to bind DNA. To determine the role of these amino acids in ssDNA binding, we expressed the mutated proteins and purified them from E. *coli*. To increase the yield of these recombinant proteins, we generated each mutated protein in an E72A background. The E72A mutation inactivates the catalytic activity of the enzyme, allowing for increased production in *E. coli* without affecting ssDNA binding. We then measured the DNA binding using two different assays. In the first assay, binding was determined using a gel retardation assay while the second assay was a fluorescence assay. In the gel retardation assay E72A, E72A/W104A, E72A/ΔWG104-5 and the E72A/RL1 chimera bound ssDNA. Interestingly, mutation of the tryptophan residues appeared to enhance ssDNA binding, suggesting that the WG104-5 insertion in A3A decreases substrate-binding affinity. The RL2 chimera and mutations of the four binding groove residues were inactive in the binding assay (Fig. 6C). Thus, the amino acid residues in the groove regulate DNA binding to A3A.

For the fluorescence-binding assay, an oligonucleotide containing a fluorescein-modified nucleotide was incubated with increasing amounts of rA3A. Fluorescence intensity measured by spectroscopy allowed us to generate a binding curve to determine the K_d_ of the interaction. The analysis showed that the E72A and the E72A/RL1 A3A bound to ssDNA with a K_d_ similar to that of wild-type. The E72A/WG104A and E72A/ΔWG104-5 proteins bound DNA with higher affinity than wild-type, consistent with the gel retardation assay results. The E72A/RL2 chimera and proteins containing point mutants in the groove lacked measurable ssDNA affinity (Fig. 6D).

### A3A is Induced by Type-I and Type-II IFN

**Figure 7:**
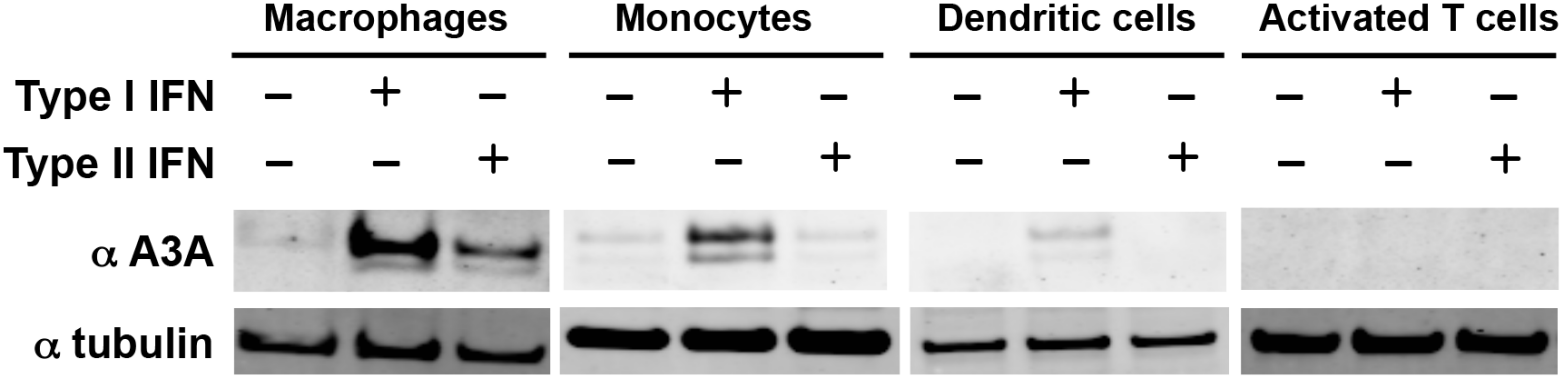
Type-I and type-II IFN induce A3A in monocytes, MDM and MDDC. Human monocytes, MDM, MDDC and activated CD4+ T cells were treated with IFN-β or IFN-γ. After 24 h the cells were lysed and protein levels determined by immunoblot using a rabbit antiserum specific for A3A. Similar results were obtained with cells purified from the blood of three independent donors.

Despite its potent cytidine deaminase activity and its ability to inhibit AAV, whether A3A has antiviral functions *in vivo* is not clear. One common feature of host proteins involved in the antiviral response is that they can be induced by type-I IFN or type-II IFN. To determine whether A3A is induced by either IFN, we utilized a polyclonal rabbit antiserum raised against the rA3A, which showed high specificity for A3A (Fig. S3). We treated primary human monocytes, MDM, MDDC and CD4^+^ T cells with type-I IFN or type-II IFN and detected the proteins on an immunoblot probed with this rabbit antiserum. This experiment confirmed the findings from Koning *et al.* [24] and Peng *et al.* [25] that type-I IFN treatment upregulates A3A protein levels in MDM and monocytes (Fig. 7). We extended these findings by demonstrating that type-I IFN treatment also slightly upregulated A3A protein levels in MDDC and that type-II IFN increased A3A protein levels in MDM. We also demonstrated that this expression is cell-type specific since neither type-I IFN nor type-II IFN treatment of CD4+ T cells upregulated A3A to a detectable level. These findings are consistent with a role for A3A in anti-viral responses in myeloid cells.

## Discussion

A detailed analysis of deamination target site preference shows that the consensus deamination sequence for A3A is TC (A/G). This finding is similar to the (T/C) CA consensus previously reported by Chen *et al*. [41] and the TC identified by others [35], [42]. We find that the A3A preferentially targets a 3′ purine. This target site preference distinguishes A3A from A3G, which targets CCC. The amino acid sequence similarity of the catalytic domains of A3A and A3G allowed us to generate chimeric proteins to determine the domains of the protein that specify the target site preference. The analysis identified a recognition loop termed RL1 in the amino-terminal domain of A3A. Replacement of RL1 with the corresponding sequence of A3G did not exchange the target site preference to CCC but allowed for deamination irrespective of the 5′ and 3′ nucleotides. Unlike A3G, A3A deaminates ssDNA substrates by a nonprocessive mechanism, consistent with the findings of Love *et al.* [35].

The mechanism by which RL1 determines target site specificity of A3A is not clear. One possibility is that residues G25 and I26 in RL1 directly contact the DNA substrate; however, the NMR structure reported by Byeon *et al*. [38] did not show an interaction between a ssDNA substrate and residues of this loop. Alternatively, the chimera may extend RL1 by three amino acids, allowing more flexibility within the substrate-binding groove and less target site constraint. This would not explain how the chimeric protein site preference is affected at both the 5′ and 3′ flanking nucleotides. Deletion of three amino acids in the RL1 of A3A in the human lineage was recently proposed to impair its antiviral activity against HIV-1 enveloped SIV [43]. The RL1 chimera, which has an insertion of three amino acids, can be packaged in HIV-1 virions but is not antiviral (data not shown), suggesting that RL1 is not solely responsible for the lack of antiviral activity of A3A against HIV-1.

Kohli *et al*. [44] have found that swapping of the recognition loop corresponding to RL2 in AID to that of A3G switched the target site preference. We found that swapping RL2 of A3G onto A3A caused the loss of deaminase activity, consistent with a similar swap reported by Narvaiza *et al*. [28]. The chimeric protein failed to bind ssDNA, accounting for its loss of catalytic activity. This finding is consistent with the NMR structure, which shows that this loop contacts the ssDNA substrate. Thus, RL2 affects target site preference but its function is influenced by other sequences in the enzyme.

Given the proximity of amino acids WG104-5 to the active site cysteine-coordinated Zn^2+^, it is surprising that insertion of these amino acids are tolerated yet deletion has no effect. In the evolution of these proteins, the insertion of these amino acid residues into A3A in new world monkeys and hominids must have occurred following the divergence of A3A and A3G. The deletion of the two amino acids caused a three-fold increase in affinity of the protein for ssDNA resulting in an affinity similar to that of A3G for its substrate. Mitra *et al.* [45] recently reported that a W104A mutation maintained catalytic activity at a reduced level while a G105A mutation resulted in a complete loss of catalytic activity. It is hard to understand how a conservative mutation at G105, where the mutated residue differs only in the presence of a methyl group, results in a complete loss of activity while a non-conservative mutation at the adjacent W104 residue did not. In light of the results of Mitra *et al*., we did an additional kinetic analysis to definitively determine the deaminase activity of the W104A A3A. The results again confirmed our finding that the W104A, RL2 and E72Q mutants lacked detectable deaminase activity (Fig. S4). Interestingly, the W104A mutant also had an increased affinity for ssDNA. This suggests that W104 plays an important role in deamination, perhaps by ensuring the proper positioning of the ssDNA substrate. This would be consistent with the A3A NMR model that indicates that W104 contacts the ssDNA substrate [38]. However, this contact must only be necessary when the adjacent G105 residue is present, as the A3G-like ΔWG104-5 mutant retains deaminase activity.

Surprisingly, the WG motif in the A3A of hominids has been replaced by a single tryptophan in Grivets, an arginine in Rhesus macaques and Colobus monkeys and deleted in new world monkeys. This motif was recently identified as being part of the ssDNA binding interface [38]. The species differences in the sequence of this loop of A3A suggests that this domain of the protein is under selective pressure. The amino acid changes could allow the protein to be less processive, thereby facilitating deamination over a larger span of viral genomes. Mutation of the tryptophan to alanine disrupted A3A catalytic activity but not ssDNA binding suggesting that the residue is involved in positioning of the ssDNA for deamination. In addition, decreased ssDNA binding affinity of A3A through mediated by alterations of the WG motif might help to prevent deamination of the cell’s genomic DNA, a problem that may be greater for A3A, which localizes to the nucleus, than other APOBEC3 proteins which are cytoplasmic. The low ssDNA binding affinity of A3A may account for its failure to deaminate retroviral DNA by causing it to localize outside of the viral core in virions [22], [23]. Lastly, the decreased affinity may provide a more dynamic interaction with ssDNA to allow it to deaminate incoming viral DNA, a feature that is unique to A3A among the APOBEC3 family members [30], [31].

The crystal structure of A3G has a groove that runs across the catalytic core of the protein that is thought to bind ssDNA [40]. Our analysis suggests a similar groove in A3A, as amino acid residues at the base of this structure affected ssDNA binding. Amino acids within the groove were essential for deaminase activity, consistent with previous reports [36], [38]. These residues are in a region of the protein that appear to contact ssDNA in the NMR structure [38], [45]. The DNA binding characteristics of A3A mutated at these residues suggests that these residues participate in DNA binding. A3A with mutations that prevent DNA binding retained the ability to be packaged into HIV-1 virions (Data not shown). A3G packaging requires an interaction with the viral genomic RNA [7], suggesting that RNA and DNA binding is mediated by different domains of the A3A. These experiments further suggest that this groove in A3A is a DNA binding site, in agreement with the recent report of Mitra *et al.* [45].

A3A has been shown to exist primarily as a monomer [35], [38], [45], [46]; however, by coimmunoprecipitation of epitope tagged A3A proteins from 293T cells and by size exclusion chromatography using *E. coli*-produced A3A we detect that a fraction of the protein was present as multimers. The detection of multimers by coimmunoprecipitation from mammalian cells suggests that they are present *in vivo*. Whether dimers might have altered biological properties is not clear. Both forms were catalytically active. Dimerization could allow for more processive deamination. It could also affect the ability of A3A to package in HIV-1 virions. Our results differed from previous reports with respect to the affinity we measured for A3A binding to ssDNA. We measured a 150 nM K_d_ while other groups measured a much lower affinity for A3A and ssDNA. We cannot explain this difference; however, A3A ssDNA binding is highly sensitive to pH [46]. It is also possible that the presence of A3A dimers in our preparations could have increased the binding affinity measured.

Despite extensive efforts, we were unable to establish a mammalian cell line that stably expressed A3A by lentiviral vector transduction while control cell-lines that expressed catalytically inactive mutated A3A were readily established (not shown). The protein as produced in *E. coli* was highly active such that even when not induced, it introduced frequent mutations in the plasmid expression vector itself. The potent deaminase activity of A3A may explain why its expression in mammals is restricted to nondividing myeloid cells and why it is induced only by specific stimuli. A3A inducibility by type-I and type-II IFNs suggests it plays an important role in antiviral defense. It has been shown to restrict human T cell leukemia virus type 1 and Rous Sarcoma Virus [27], [47] but it is unclear as to whether these are its natural targets. A precise definition of target site preference will be useful in identifying A3A-generated mutations in pathogens. In addition, the ability to alter the target site preference of A3A may allow for tailoring the protein to alter its ability to restrict the replication of pathogens.

## Materials and Methods

### Cell Culture

293T and HeLa cells were cultured in Dulbecco’s modified Eagle medium supplemented with 10% fetal bovine serum and penicillin and streptomycin. Primary monocytes, monocyte-derived macrophages (MDM), monocyte-derived dendritic cells (MDDC) and T cells were cultured in RPMI 1640 supplemented with 10% FBS and penicillin and streptomycin. Monocytes were purified from leukocyte enriched blood samples obtained from anonymous donors by the New York Blood Center using anti-CD14-conjugated magnetic beads (Miltenyi Biotec) and differentiated into MDM by culturing for 7 days with 50 ng/mL GM-CSF or into MDDC by culturing with 50 ng/mL GM-CSF and 100 ng/mL IL-4 (Invitrogen). MDM and MDDCs were plated in 12 well plates at 1.0×10^6^ cells per well and cultured for 20 h with or without 2000 units of Universal Type I IFN (PBL Biomedical Laboratories) or 2.0 µg/ml γ-IFN after which they were lysed in protease-supplemented lysis buffer (50 mM Tris pH 7.5, 150 mM NaCl, 2 mM EDTA and 1% NP40).

### Plasmids

Mammalian expression vectors for FLAG-tagged, HA-tagged, and Myc-His tagged A3A were generated by PCR amplification of the coding sequence with oligonucleotide primers containing EcoR-I and Xho-I sites and ligated to pcDNA6 A/myc-his (Invitrogen). Amplicon lacking a Kozak sequence was cloned into pET42a+ to generate the pET42.A3A for expression of a GST-A3A fusion protein in *E. coli*. Point mutations were introduced into A3A by overlapping PCR. All plasmids were confirmed by nucleotide sequencing and expression of the encoded proteins was confirmed by immunoblot analysis.

### Recombinant A3A Expression and Purification

*E. coli* BL21 (DE3) CodonPlus RIPL cells (Stratagene) were transformed with pET42a-A3A and single colonies were cultured overnight and used to seed 500 ml of Luria broth. The cultures were grown for 5 h at 37°C and induced for 2 h with 200 µM IPTG. The bacteria were pelleted, freeze thawed, resuspended in PBS, sonicated and lysed in buffer containing 1% Triton-X100. The lysate was treated for 1 h at rm. temp. with 25 units/ml of Benzonuclease (Novagen) and then clarified by centrifugation for 10 min at 6000×g. The recombinant protein was collected on 500 µl of glutathione agarose (Sigma) for 16 h at 4°C. The resin was washed with PBS and resuspended in buffer containing 50 mM Tris-HCl (pH 8.0), 100 mM NaCl, 5 mM CaCl_2_ (pH 8.0). The protein was cleaved from the beads with 10 U Factor Xa (Novagen) and used for size exclusion chromatography or concentrated on YM-10 (Millipore) and stored at −20°C in 50% glycerol. rA3A, without concentration, was analyzed by FPLC on a Superose-12 gel filtration column (GE) with protein standards. 1.0 ml fractions were collected and the protein concentration and deaminase activity was determined for each fraction.

### Mammalian A3A Expression

293T cells (5.0×10^5^) were transfected with 4.0 µg pcDNA6-A3A-myc-his using Lipofectamine 2000 (Invitrogen). After 48 h, the cells were lysed in lysis buffer (50 mM Tris pH 8.0, 40 mM KCl, 50 mM NaCl, 5 mM EDTA, 0.1% Triton-X100, 10 mM DTT, protease inhibitors). To immunoprecipitate A3A, the lysates were precleared with protein-G sepharose (GE Healthcare) and immunoprecipitated with anti-myc antibody (Covance) and protein-G sepharose for 2 h at 4°C. Unbound protein was removed by washing and the beads were resuspended in deaminase buffer (40 mM Tris pH 8.0, 10% glycerol, 40 mM KCl, 50 mM NaCl, 5 mM EDTA, 5 µM DTT).

### *In vitro* Deaminase Assay

*E. coli* expressed A3A (1.0 µg), FPLC fractions (10 µl) or mammalian expressed A3A bound on sepharose beads (5 µl) was incubated with 10 pmoles of 5′-biotin-labeled oligonucleotide in deaminase buffer for 60 min (*E. coli* A3A), or 1–6 h (mammalian A3A) at 37°C. The A3A was inactivated for 3 min at 95°C and then treated for 30 min at 37°C with 1 unit UDG in buffer containing 20 mM Tris pH 8.0, 1 mM DTT, 1 mM EDTA. The product was hydrolyzed for 30 min in 140 mM NaOH at 37°C. The resulting oligonucleotide fragments were separated on a 15% TBE-urea PAGE gel and transferred to a nylon filter (Invitrogen). The membranes were UV crosslinked, blocked with 5% milk and incubated for 30 min. with 1.0 µg of DyLight 800 conjugated Streptavidin (Pierce). Signals were quantified on an Odyssey Imager (LI-COR). The percent deamination was defined as the amount of cleaved oligonucleotide divided by the sum of the cleaved and uncleaved oligonucleotides.

### Substrates

Most of the deaminase assays used a biotinylated oligonucleotide with a single TCA site (5′-biotin-T_28_(TCA)T_28_-3′) (Integrated DNA Technologies). To determine the deamination site preference of rA3A, a set of biotinylated oligos containing all possible three-base combinations were used (5′-biotin-T_14_(ACA)T_7_(ACT)T_9_(ACG)T_17_(ACC)T_31_-3′, 5′-biotin-T_14_(TCA)T_7_(TCT)T_9_(TCG)T_17_(TCC)T_31_-3′, 5′-biotin-T_14_(CCA)T_7_(CCT)T_9_(CCG)T_17_(CCC)T_31_-3′, 5′-biotin-T_14_(GCA)T_7_(GCT)T_9_(GCG)T_17_(GCC)T_31_-3′). Trinucleotide deaminase specificity was determined using biotinylated oligos with each possible trinucleotide context: NCN sites (5′-biotin-T_28_(NCN)T_28_-3′).

### Deamination Sequencing

An oligonucleotide containing repeating rA3A preferred deamination sites (5′-biotin-GGG GGT AGA TTG AGG TAA GTA (TCA)_18_TGA ATA GGA GTG TTA AGG GGG-3′) was treated with 50 ng rA3A in deaminase assay buffer. The deaminated DNA was cloned into TOPO TA (Invitrogen) and sequenced.

### Anti-A3A Antiserum Production and Immunoblot Analysis

Anti-A3A antiserum was raised at Pocono Rabbit Farm & Laboratory (PRF&L) by repeated immunization of 2 rabbits with *E. coli*-produced rA3A from which the GST had been removed. Protein lysates (30 µg) were separated on a 4–12% SDS PAGE gradient gel and then transferred to a PVDF membrane. The membrane was blocked in 5% milk and incubated with 1∶1000 anti-A3A serum diluted, 1∶5000 anti-α-tubulin monoclonal antibody B-5-1-2 (Sigma) or 1∶1000 anti-Myc monoclonal antibody 9E10 (Covance). Bound antibody was detected by hybridizing to 1∶1000 anti-rabbit or anti-mouse biotin-conjugated secondary antibody followed by streptavidin conjugated DyLight 800 (Thermo Scientific) and visualized on an Odyssey Infrared Imaging System (LI-COR Biosciences).

### DNA Binding Assays

DNA binding by electrophoretic mobility shift assay was determined by incubating 0.5 pmol of 5′-fluorescein-conjugated (T)_8_CG(T)_6 _DNA oligonucleotide at 4°C with 2 µg rA3A in 10 µl deaminase buffer for 1 h. The samples were separated by 6% native PAGE and visualized on a Typhoon Trio imager (GE Healthcare). DNA binding was quantified by measuring the intrinsic fluorescence of 225 nM rA3A with increasing amounts of oligodeoxynucleotide in 25 mM Tris pH 8.0 and 25 mM NaCl on a FluoroMax-4 spectrofluorimeter [28], [36]. The samples were excited at 295 nm and emission was detected at 345–355 nm. The extrinsic fluorescence of a fluorescein-conjugated ssDNA oligodeoxynucleotide with an increasing concentration of rA3A was measured in triplicate on a Perkin Elmer Envision plate reader. The samples were excited at 485 nm and emission was detected at 535 nm. The change in intrinsic and extrinsic fluorescence was plotted against the concentration of the unlabeled oligodeoxynucleotide and rA3A respectively using SigmaPlot software. A one-site saturation ligand-binding curve was fitted for each plot and the K_d_ was determined based on the ligand-binding curve.

### A3A and A3G Structure Analysis

A3A NMR structure (2M65, [38]) and A3G catalytic domain crystal structure (3E1U, [39]) were analyzed using PyMOL software.

### Ethics Statement

Anti-A3A antiserum was prepared by Pocono Rabbit Farm and Laboratories (PRF&L) under the protocol PRF2A approved by PRF&L Institutional Animal Care and Use Committee (IACUC). PRF&L is accredited by the Association for Assessment and Accreditation of Laboratory Animal Care (AAALAC) and by the National Institutes of Health (NIH) Office of Laboratory Animal Welfare (OLAW), assurance number A3886-01 expiration January 31, 2017.

## Supporting Information

Figure S1 **rA3A monomer and dimer fraction are catalytically active.** Deaminase activity of 1.0 µg A3A protein extract or 10 µl of each size exclusion chromatography fraction was determined by incubation with an oligonucleotide containing a TCA consensus target sequence. The results are representative of two independent repetitions using different batches of rA3A.
(TIF)

Figure S2 **Comparative A3A and A3G structure.** A) Alignment of A3A and the carboxy terminal catalytic domain of A3G primary sequence against A3G secondary structure. The sequences were aligned using ClustalW2 (www.ebi.ac.uk/Tools/msa/clustalw2/). Identical amino acids are in white on a red background. A3G secondary structure was extracted from its crystal structure. The α-helices are represented above their corresponding primary sequence by a drawn helix, while arrows represent β-sheets. Each residue mutated in A3A is indicated with a triangle containing the matched color from the 3D model of Figures 5 and 6. A black bar below the sequence delimits RL1 and 2. B) A 3D model of A3A displaying the tertiary structure is displayed. For each amino acid mutated or swapped loop, an arrow indicates its position.
(TIF)

Figure S3 **The rabbit anti-A3A antibody does not cross-react with other members of the APOBEC3 family.** 293T were transfected with an empty pcDNA6 vector (EV) or vectors encoding HA-tagged A3A, APOBEC3B, APOBEC3C, A3F and A3G. Lysates were separated by SDS-PAGE, and blotted with mouse anti-HA antibody, to control for protein expression, or a rabbit antiserum raised against rA3A.
(TIF)

Figure S4 **W104A is catalytically inactive.** Wild-type and mutated A3A were expressed in transfected 293T cells, immunoprecipitated from cell lysates and then tested for deaminase activity against an oligonucleotide containing a TCG target sequence. The activity was measured at the indicated time points.
(TIF)

## References

[1] Conticello SG (2008) The AID/APOBEC family of nucleic acid mutators. Genome Biol 9: 229. 10.1186/gb-2008-9-6-229 PMC2481415 18598372

[2] Jarmuz A, Chester A, Bayliss J, Gisbourne J, Dunham I, et al (2002) An anthropoid-specific locus of orphan C to U RNA-editing enzymes on chromosome 22. Genomics 79: 285–296. 11863358 10.1006/geno.2002.6718

[3] Wedekind JE, Dance GS, Sowden MP, Smith HC (2003) Messenger RNA editing in mammals: new members of the APOBEC family seeking roles in the family business. Trends Genet 19: 207–216. 12683974 10.1016/S0168-9525(03)00054-4

[4] Alce TM, Popik W (2004) APOBEC3G is incorporated into virus-like particles by a direct interaction with HIV-1 Gag nucleocapsid protein. J Biol Chem 279: 34083–34086. 15215254 10.1074/jbc.C400235200

[5] Cen S, Guo F, Niu M, Saadatmand J, Deflassieux J, et al (2004) The interaction between HIV-1 Gag and APOBEC3G. J Biol Chem 279: 33177–33184. 15159405 10.1074/jbc.M402062200

[6] Douaisi M, Dussart S, Courcoul M, Bessou G, Vigne R, et al (2004) HIV-1 and MLV Gag proteins are sufficient to recruit APOBEC3G into virus-like particles. Biochem Biophys Res Commun 321: 566–573. 15358144 10.1016/j.bbrc.2004.07.005

[7] Khan MA, Kao S, Miyagi E, Takeuchi H, Goila-Gaur R, et al (2005) Viral RNA is required for the association of APOBEC3G with human immunodeficiency virus type 1 nucleoprotein complexes. J Virol 79: 5870–5874. 15827203 10.1128/JVI.79.9.5870-5874.2005 PMC1082784

[8] Luo K, Liu B, Xiao Z, Yu Y, Yu X, et al (2004) Amino-terminal region of the human immunodeficiency virus type 1 nucleocapsid is required for human APOBEC3G packaging. J Virol 78: 11841–11852. 15479826 10.1128/JVI.78.21.11841-11852.2004 PMC523292

[9] Navarro F, Bollman B, Chen H, Konig R, Yu Q, et al (2005) Complementary function of the two catalytic domains of APOBEC3G. Virology 333: 374–386. 15721369 10.1016/j.virol.2005.01.011

[10] Schafer A, Bogerd HP, Cullen BR (2004) Specific packaging of APOBEC3G into HIV-1 virions is mediated by the nucleocapsid domain of the gag polyprotein precursor. Virology 328: 163–168. 15464836 10.1016/j.virol.2004.08.006

[11] Svarovskaia ES, Xu H, Mbisa JL, Barr R, Gorelick RJ, et al (2004) Human apolipoprotein B mRNA-editing enzyme-catalytic polypeptide-like 3G (APOBEC3G) is incorporated into HIV-1 virions through interactions with viral and nonviral RNAs. J Biol Chem 279: 35822–35828. 15210704 10.1074/jbc.M405761200

[12] Wang T, Tian C, Zhang W, Luo K, Sarkis PT, et al (2007) 7SL RNA mediates virion packaging of the antiviral cytidine deaminase APOBEC3G. J Virol 81: 13112–13124. 17881443 10.1128/JVI.00892-07 PMC2169093

[13] Wang T, Zhang W, Tian C, Liu B, Yu Y, et al (2008) Distinct viral determinants for the packaging of human cytidine deaminases APOBEC3G and APOBEC3C. Virology 377: 71–79. 18495196 10.1016/j.virol.2008.04.012 PMC2692739

[14] Zennou V, Perez-Caballero D, Gottlinger H, Bieniasz PD (2004) APOBEC3G incorporation into human immunodeficiency virus type 1 particles. J Virol 78: 12058–12061. 15479846 10.1128/JVI.78.21.12058-12061.2004 PMC523273

[15] Harris RS, Bishop KN, Sheehy AM, Craig HM, Petersen-Mahrt SK, et al (2003) DNA deamination mediates innate immunity to retroviral infection. Cell 113: 803–809. 12809610 10.1016/s0092-8674(03)00423-9

[16] Lecossier D, Bouchonnet F, Clavel F, Hance AJ (2003) Hypermutation of HIV-1 DNA in the absence of the Vif protein. Science 300: 1112. 12750511 10.1126/science.1083338

[17] Mangeat B, Turelli P, Caron G, Friedli M, Perrin L, et al (2003) Broad antiretroviral defence by human APOBEC3G through lethal editing of nascent reverse transcripts. Nature 424: 99–103. 12808466 10.1038/nature01709

[18] Yu Q, Konig R, Pillai S, Chiles K, Kearney M, et al (2004) Single-strand specificity of APOBEC3G accounts for minus-strand deamination of the HIV genome. Nat Struct Mol Biol 11: 435–442. 15098018 10.1038/nsmb758

[19] Zhang H, Yang B, Pomerantz RJ, Zhang C, Arunachalam SC, et al (2003) The cytidine deaminase CEM15 induces hypermutation in newly synthesized HIV-1 DNA. Nature 424: 94–98. 12808465 10.1038/nature01707 PMC1350966

[20] Sheehy AM, Gaddis NC, Choi JD, Malim MH (2002) Isolation of a human gene that inhibits HIV-1 infection and is suppressed by the viral Vif protein. Nature 418: 646–650. 12167863 10.1038/nature00939

[21] Chen H, Lilley CE, Yu Q, Lee DV, Chou J, et al (2006) APOBEC3A is a potent inhibitor of adeno-associated virus and retrotransposons. Curr Biol 16: 480–485. 16527742 10.1016/j.cub.2006.01.031

[22] Aguiar RS, Lovsin N, Tanuri A, Peterlin BM (2008) Vpr.A3A chimera inhibits HIV replication. J Biol Chem 283: 2518–2525. 18057006 10.1074/jbc.M706436200

[23] Goila-Gaur R, Khan MA, Miyagi E, Kao S, Strebel K (2007) Targeting APOBEC3A to the viral nucleoprotein complex confers antiviral activity. Retrovirology 4: 61. 17727729 10.1186/1742-4690-4-61 PMC2018723

[24] Koning FA, Goujon C, Bauby H, Malim MH (2011) Target cell-mediated editing of HIV-1 cDNA by APOBEC3 proteins in human macrophages. J Virol 85: 13448–13452. 21957290 10.1128/JVI.00775-11 PMC3233168

[25] Peng G, Greenwell-Wild T, Nares S, Jin W, Lei KJ, et al (2007) Myeloid differentiation and susceptibility to HIV-1 are linked to APOBEC3 expression. Blood 110: 393–400. 17371941 10.1182/blood-2006-10-051763 PMC1896122

[26] Stenglein MD, Burns MB, Li M, Lengyel J, Harris RS (2010) APOBEC3 proteins mediate the clearance of foreign DNA from human cells. Nat Struct Mol Biol 17: 222–229. 20062055 10.1038/nsmb.1744 PMC2921484

[27] Ooms M, Krikoni A, Kress AK, Simon V, Munk C (2012) APOBEC3A, APOBEC3B, and APOBEC3H haplotype 2 restrict human T-lymphotropic virus type 1. J Virol 86: 6097–6108. 22457529 10.1128/JVI.06570-11 PMC3372211

[28] Narvaiza I, Linfesty DC, Greener BN, Hakata Y, Pintel DJ, et al (2009) Deaminase-independent inhibition of parvoviruses by the APOBEC3A cytidine deaminase. PLoS Pathog 5: e1000439. 19461882 10.1371/journal.ppat.1000439 PMC2678267

[29] Bogerd HP, Wiegand HL, Doehle BP, Lueders KK, Cullen BR (2006) APOBEC3A and APOBEC3B are potent inhibitors of LTR-retrotransposon function in human cells. Nucleic Acids Res 34: 89–95. 16407327 10.1093/nar/gkj416 PMC1326241

[30] Berger A, Munk C, Schweizer M, Cichutek K, Schule S, et al (2010) Interaction of Vpx and apolipoprotein B mRNA-editing catalytic polypeptide 3 family member A (APOBEC3A) correlates with efficient lentivirus infection of monocytes. J Biol Chem 285: 12248–12254. 20178977 10.1074/jbc.M109.090977 PMC2852964

[31] Berger G, Durand S, Fargier G, Nguyen XN, Cordeil S, et al (2011) APOBEC3A is a specific inhibitor of the early phases of HIV-1 infection in myeloid cells. PLoS Pathog 7: e1002221. 21966267 10.1371/journal.ppat.1002221 PMC3178557

[32] Land AM, Law EK, Carpenter MA, Lackey L, Brown WL, et al (2013) Endogenous APOBEC3A DNA cytosine deaminase is cytoplasmic and nongenotoxic. J Biol Chem 288: 17253–17260. 23640892 10.1074/jbc.M113.458661 PMC3682529

[33] Landry S, Narvaiza I, Linfesty DC, Weitzman MD (2011) APOBEC3A can activate the DNA damage response and cause cell-cycle arrest. EMBO Rep 12: 444–450. 21460793 10.1038/embor.2011.46 PMC3090015

[34] Chelico L, Pham P, Calabrese P, Goodman MF (2006) APOBEC3G DNA deaminase acts processively 3′–>5′ on single-stranded DNA. Nat Struct Mol Biol 13: 392–399. 16622407 10.1038/nsmb1086

[35] Love RP, Xu H, Chelico L (2012) Biochemical analysis of hypermutation by the deoxycytidine deaminase APOBEC3A. J Biol Chem 287: 30812–30822. 22822074 10.1074/jbc.M112.393181 PMC3436324

[36] Bulliard Y, Narvaiza I, Bertero A, Peddi S, Rohrig UF, et al (2011) Structure-function analyses point to a polynucleotide-accommodating groove essential for APOBEC3A restriction activities. J Virol 85: 1765–1776. 21123384 10.1128/JVI.01651-10 PMC3028873

[37] Ellinger P, Arslan Z, Wurm R, Tschapek B, MacKenzie C, et al (2012) The crystal structure of the CRISPR-associated protein Csn2 from Streptococcus agalactiae. J Struct Biol 178: 350–362. 22531577 10.1016/j.jsb.2012.04.006

[38] Byeon IJ, Ahn J, Mitra M, Byeon CH, Hercik K, et al (2013) NMR structure of human restriction factor APOBEC3A reveals substrate binding and enzyme specificity. Nat Commun 4: 1890. 23695684 10.1038/ncomms2883 PMC3674325

[39] Holden LG, Prochnow C, Chang YP, Bransteitter R, Chelico L, et al (2008) Crystal structure of the anti-viral APOBEC3G catalytic domain and functional implications. Nature 456: 121–124. 18849968 10.1038/nature07357 PMC2714533

[40] Chen KM, Harjes E, Gross PJ, Fahmy A, Lu Y, et al (2008) Structure of the DNA deaminase domain of the HIV-1 restriction factor APOBEC3G. Nature 452: 116–119. 18288108 10.1038/nature06638

[41] Levin MK, Gurjar M, Patel SS (2005) A Brownian motor mechanism of translocation and strand separation by hepatitis C virus helicase. Nat Struct Mol Biol 12: 429–435. 15806107 10.1038/nsmb920

[42] Thielen BK, McNevin JP, McElrath MJ, Hunt BV, Klein KC, et al (2010) Innate immune signaling induces high levels of TC-specific deaminase activity in primary monocyte-derived cells through expression of APOBEC3A isoforms. J Biol Chem 285: 27753–27766. 20615867 10.1074/jbc.M110.102822 PMC2934643

[43] Schmitt K, Guo K, Algaier M, Ruiz A, Cheng F, et al (2011) Differential virus restriction patterns of rhesus macaque and human APOBEC3A: implications for lentivirus evolution. Virology 419: 24–42. 21868050 10.1016/j.virol.2011.07.017 PMC4104698

[44] Kohli RM, Abrams SR, Gajula KS, Maul RW, Gearhart PJ, et al (2009) A portable hot spot recognition loop transfers sequence preferences from APOBEC family members to activation-induced cytidine deaminase. J Biol Chem 284: 22898–22904. 19561087 10.1074/jbc.M109.025536 PMC2755697

[45] Mitra M, Hercik K, Byeon IJ, Ahn J, Hill S, et al (2014) Structural determinants of human APOBEC3A enzymatic and nucleic acid binding properties. Nucleic Acids Res 42: 1095–1110. 24163103 10.1093/nar/gkt945 PMC3902935

[46] Pham P, Landolph A, Mendez C, Li N, Goodman MF (2013) A biochemical analysis linking APOBEC3A to disparate HIV-1 restriction and skin cancer. J Biol Chem 288: 29294–29304. 23979356 10.1074/jbc.M113.504175 PMC3795231

[47] Wiegand HL, Cullen BR (2007) Inhibition of alpharetrovirus replication by a range of human APOBEC3 proteins. J Virol 81: 13694–13699. 17913830 10.1128/JVI.01646-07 PMC2168862

