# Supplementary figures and images for: A DNA Sequence Recognition Loop on APOBEC3A Controls Substrate Specificity
Source: PLoS One. 2014 May 14;9(5):e97062. doi: 10.1371/journal.pone.0097062 (PMC4020817; doi:10.1371/journal.pone.0097062)

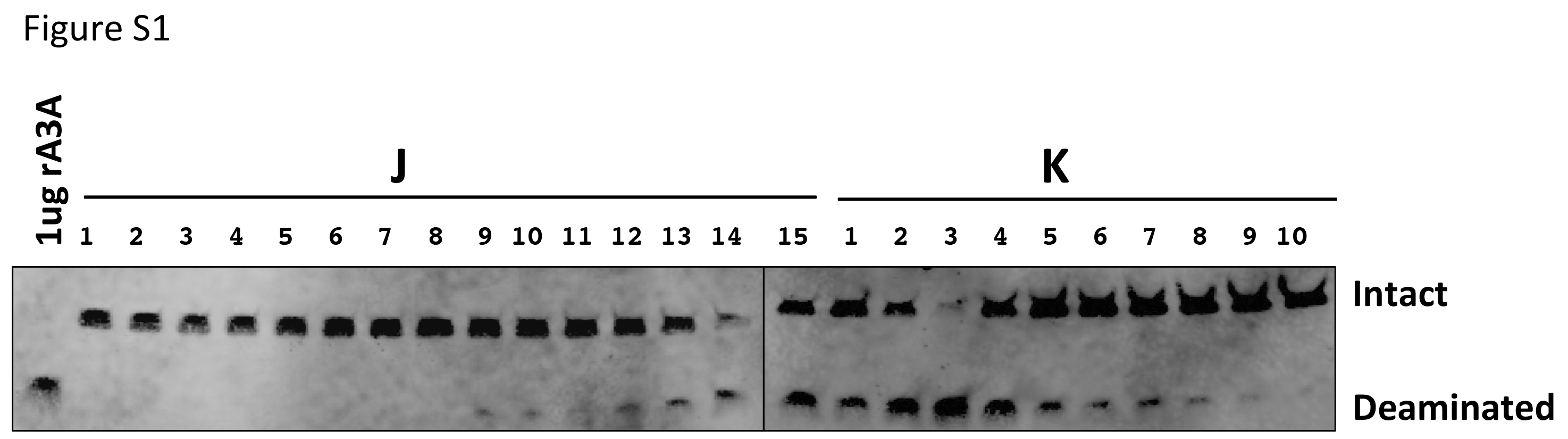

Supplement: Figure S1 — rA3A monomer and dimer fraction are catalytically active. Deaminase activity of 1.0 µg A3A protein extract or 10 µl of each size exclusion chromatography fraction was determined by incubation with an oligonucleotide containing a TCA consensus target sequence. The results are representative of two independent repetitions using different batches of rA3A. (TIF) [file pone.0097062.s001.tif]

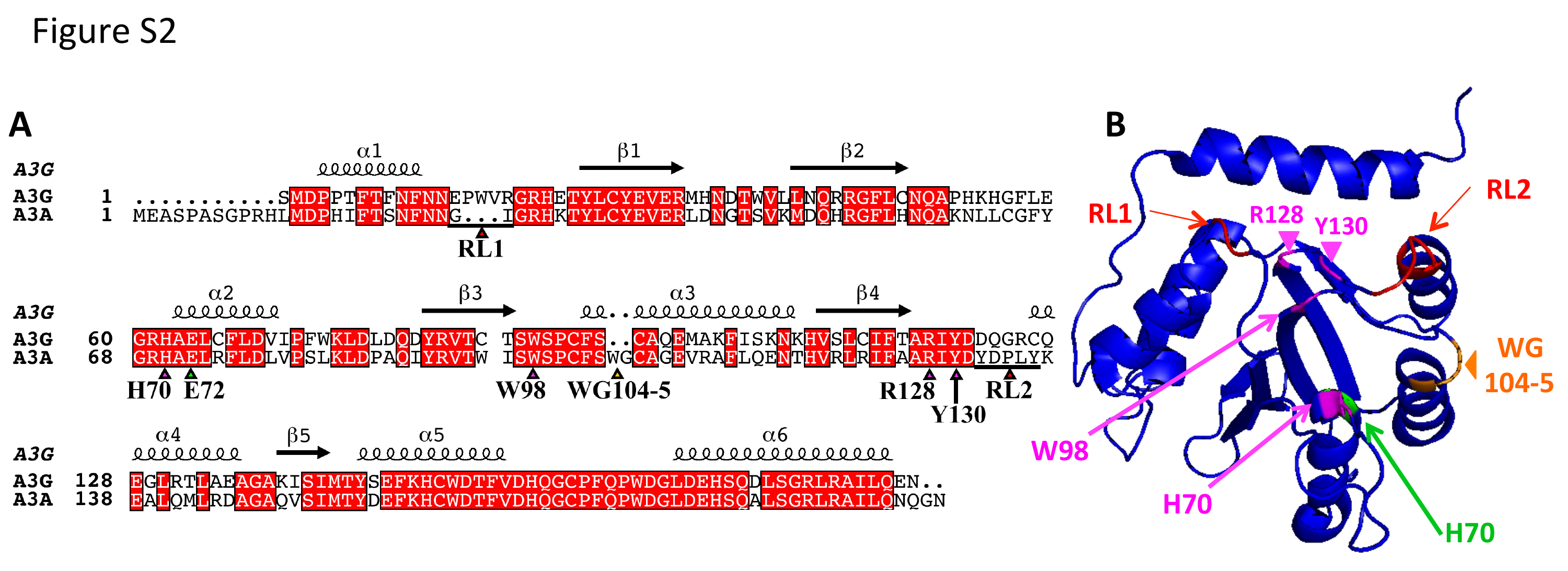

Supplement: Figure S2 — Comparative A3A and A3G structure. A) Alignment of A3A and the carboxy terminal catalytic domain of A3G primary sequence against A3G secondary structure. The sequences were aligned using ClustalW2 (www.ebi.ac.uk/Tools/msa/clustalw2/). Identical amino acids are in white on a red background. A3G secondary structure was extracted from its crystal structure. The α-helices are represented above their corresponding primary sequence by a drawn helix, while arrows represent β-sheets. Each residue mutated in A3A is indicated with a triangle containing the matched color from the 3D model of Figures 5 and 6. A black bar below the sequence delimits RL1 and 2. B) A 3D model of A3A displaying the tertiary structure is displayed. For each amino acid mutated or swapped loop, an arrow indicates its position. (TIF) [file pone.0097062.s002.tif]

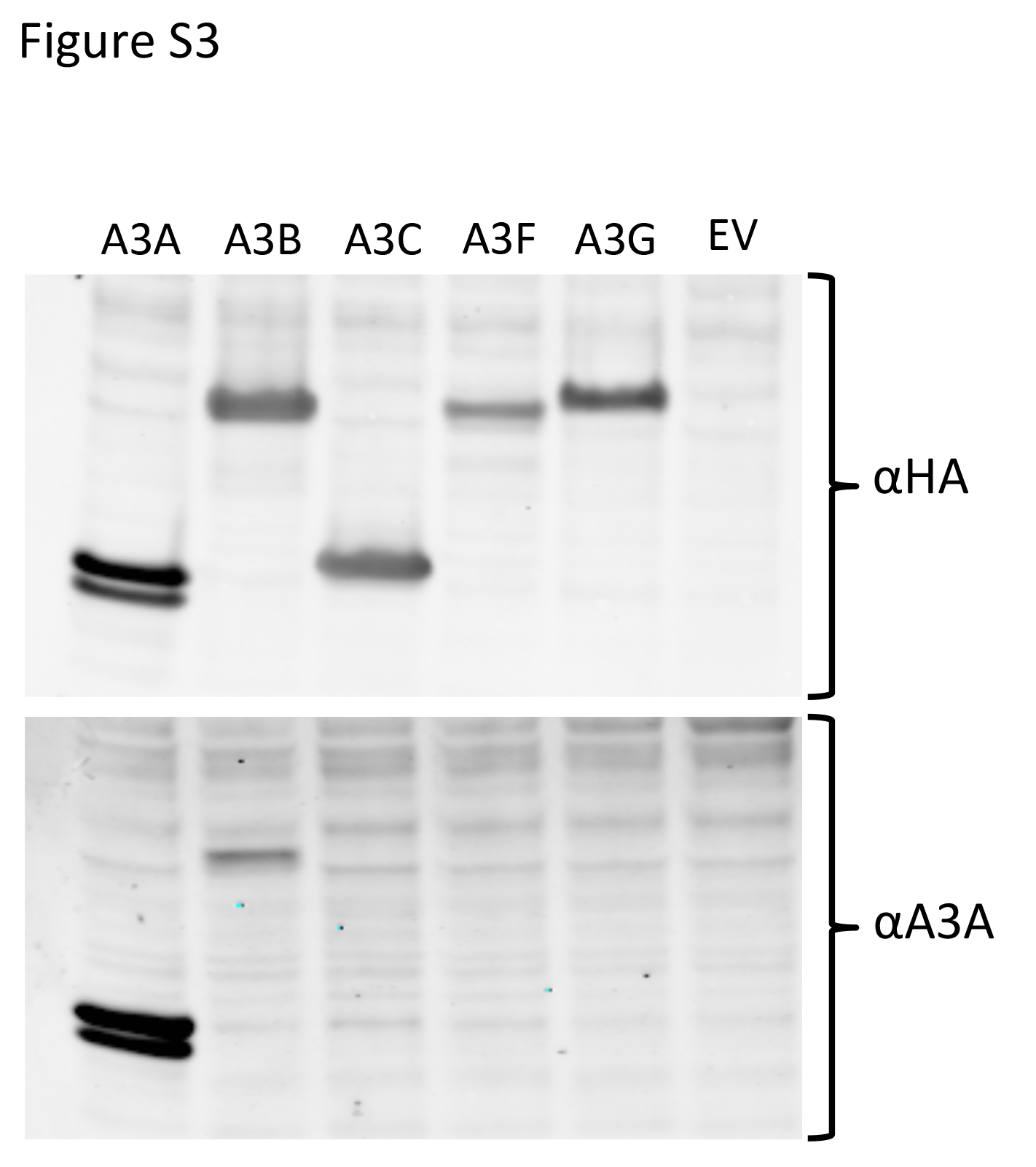

Supplement: Figure S3 — The rabbit anti-A3A antibody does not cross-react with other members of the APOBEC3 family. 293T were transfected with an empty pcDNA6 vector (EV) or vectors encoding HA-tagged A3A, APOBEC3B, APOBEC3C, A3F and A3G. Lysates were separated by SDS-PAGE, and blotted with mouse anti-HA antibody, to control for protein expression, or a rabbit antiserum raised against rA3A. (TIF) [file pone.0097062.s003.tif]

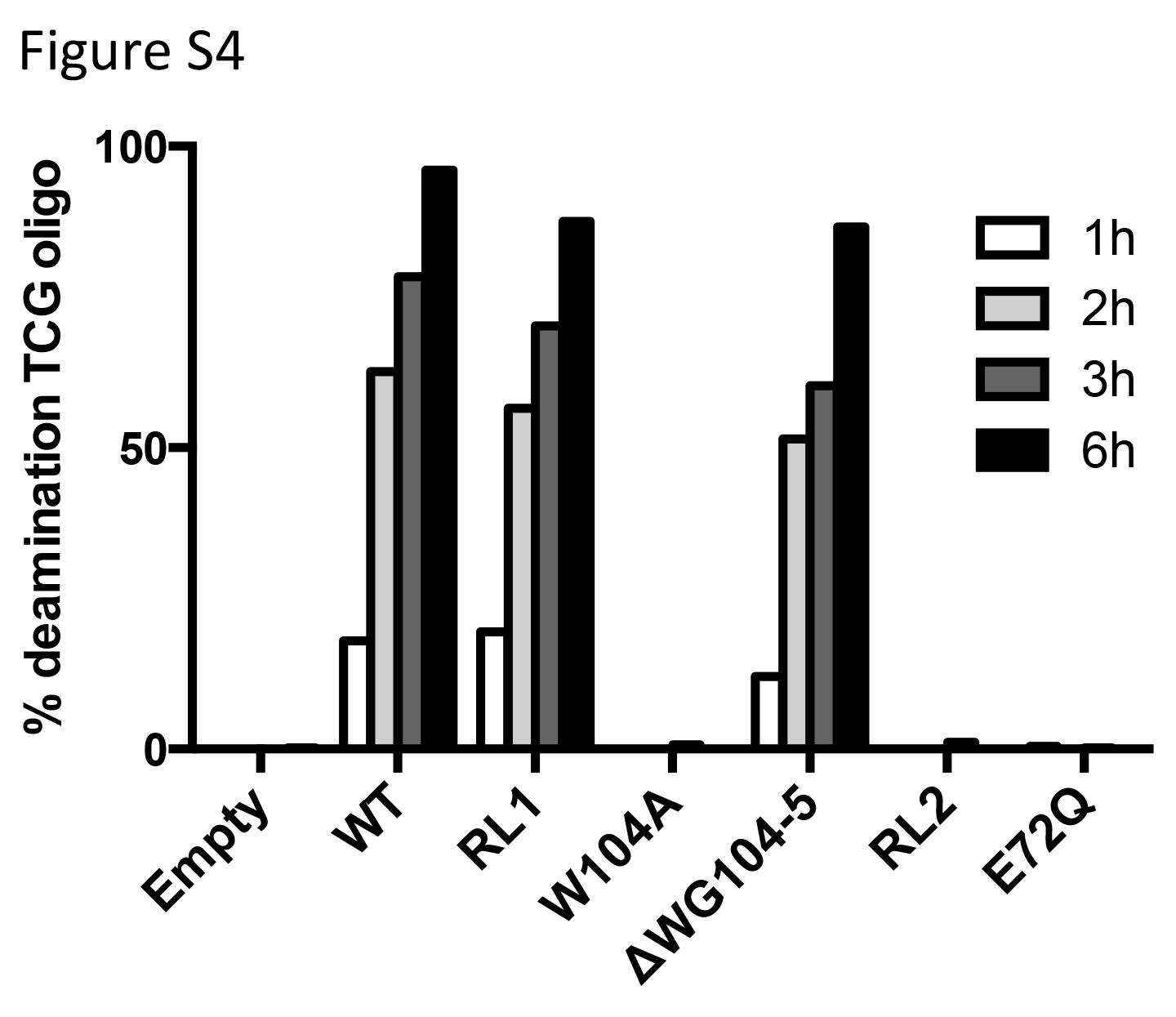

Supplement: Figure S4 — W104A is catalytically inactive. Wild-type and mutated A3A were expressed in transfected 293T cells, immunoprecipitated from cell lysates and then tested for deaminase activity against an oligonucleotide containing a TCG target sequence. The activity was measured at the indicated time points. (TIF) [file pone.0097062.s004.tif]
